# XAV-19, a Swine Glyco-Humanized Polyclonal Antibody Against SARS-CoV-2 Spike Receptor-Binding Domain, Targets Multiple Epitopes and Broadly Neutralizes Variants

**DOI:** 10.3389/fimmu.2021.761250

**Published:** 2021-11-15

**Authors:** Bernard Vanhove, Stéphane Marot, Ray T. So, Benjamin Gaborit, Gwénaëlle Evanno, Isabelle Malet, Guillaume Lafrogne, Edwige Mevel, Carine Ciron, Pierre-Joseph Royer, Elsa Lheriteau, François Raffi, Roberto Bruzzone, Chris Ka Pun Mok, Odile Duvaux, Anne-Geneviève Marcelin, Vincent Calvez

**Affiliations:** ^1^ Xenothera, Nantes, France; ^2^ Sorbonne Université, Institut National de la Santé et de la Recherche Médicale (INSERM) 1136, Institut Pierre Louis d’Epidémiologie et de Santé Publique (iPLESP), Assistance Publique-Hôpitaux de Paris (AP-HP), Pitié Salpêtrière Hospital, Department of Virology, Paris, France; ^3^ Hong Kong University (HKU)-Pasteur Research Pole, School of Public Health, Li Ka Shing (LKS) Faculty of Medicine, The University of Hong Kong, Hong Kong, Hong Kong SAR, China; ^4^ Department of Infectious Disease, Nantes University Hospital, Nantes, France; ^5^ Institut National de la Santé et de la Recherche Médicale (INSERM) CIC1413, Nantes University Hospital, Nantes, France; ^6^ Department of Cell Biology and Infection, Institut Pasteur, Paris, France; ^7^ Li Ka Shing Institute of Health Sciences, Faculty of Medicine, The Chinese University of Hong Kong, Hong Kong, Hong Kong SAR, China; ^8^ The Jockey Club School of Public Health and Primary Care, The Chinese University of Hong Kong, Hong Kong, Hong Kong SAR, China

**Keywords:** COVID-19, polyclonal antibody (PAb), SARS-CoV-2, variants, neutralization

## Abstract

Amino acid substitutions and deletions in the Spike protein of severe acute respiratory syndrome coronavirus 2 (SARS-CoV-2) variants can reduce the effectiveness of monoclonal antibodies (mAbs). In contrast, heterologous polyclonal antibodies raised against S protein, through the recognition of multiple target epitopes, have the potential to maintain neutralization capacities. XAV-19 is a swine glyco-humanized polyclonal neutralizing antibody raised against the receptor binding domain (RBD) of the Wuhan-Hu-1 Spike protein of SARS-CoV-2. XAV-19 target epitopes were found distributed all over the RBD and particularly cover the receptor binding motives (RBMs), in direct contact sites with the angiotensin converting enzyme-2 (ACE-2). Therefore, in Spike/ACE-2 interaction assays, XAV-19 showed potent neutralization capacities of the original Wuhan Spike and of the United Kingdom (Alpha/B.1.1.7) and South African (Beta/B.1.351) variants. These results were confirmed by cytopathogenic assays using Vero E6 and live virus variants including the Brazil (Gamma/P.1) and the Indian (Delta/B.1.617.2) variants. In a selective pressure study on Vero E6 cells conducted over 1 month, no mutation was associated with the addition of increasing doses of XAV-19. The potential to reduce viral load in lungs was confirmed in a human ACE-2 transduced mouse model. XAV-19 is currently evaluated in patients hospitalized for COVID-19-induced moderate pneumonia in phase 2a-2b (NCT04453384) where safety was already demonstrated and in an ongoing 2/3 trial (NCT04928430) to evaluate the efficacy and safety of XAV-19 in patients with moderate-to-severe COVID-19. Owing to its polyclonal nature and its glyco-humanization, XAV-19 may provide a novel safe and effective therapeutic tool to mitigate the severity of coronavirus disease 2019 (COVID-19) including the different variants of concern identified so far.

## Introduction

Passive antibody therapies have demonstrated efficacy to reduce the progression of mild coronavirus disease 2019 (COVID-19) to severe disease if administered early enough in the course of illness (1–3). Three sources of antibodies have so far been assessed. First, passive antibody therapy using the infusion of convalescent plasma (CP) with high SARS-CoV-2 antibody titers in hospitalized patients, administered within 72 h after the onset of mild symptoms, reduced the relative risk of progression to severe disease by 73% if CP presented a titer of >1:3,200 and by 31.4% with lower titer CP (1). This was true with CP drawn between June and October 2020. However, the CP from patients infected by the original SARS-CoV-2 lineage had poor activity against the Beta variant, and this was attributed to three mutations (K417N, E484K, and N501Y) in the Spike protein (4). Among these mutations, E484K has been shown to play a major role in reducing the binding and neutralization (5). Second, besides the use of CP, more than 50 neutralizing monoclonal antibodies (mAbs) are in development against the Spike receptor-binding domain (RBD) and the N-terminal domain (NTD) of SARS-CoV-2 (6). Those developed by Regeneron Pharmaceuticals (REGN-COV2 (casirivimab/imdevimab cocktail), Eli Lilly (bamlanivimab/etesevimab), Celltrion (regdanvimab), and GSK (sotrovimab) provide protection against the risk of severe COVID-19 when administered early in high-risk symptomatic patients with mild to moderate COVID-19 not requiring hospitalization (7). However, viral mutations can escape the mAbs, which are used to treat the infection of SARS-CoV-2 (8). The Alpha variant is refractory to neutralization by most mAbs, which target the NTD of Spike protein and also resistant to several RBD-specific mAbs (9). Mutations in the Beta lineage (K417N, E484K, and N501Y in RBD), especially mutations of Spike at E484 but also in the N-terminal domain (NTD; L18F, D80A, D215G, Δ242-244, and R246I in SA variant (10, 11)), reduce neutralization sensitivity or confer neutralization escape from multiple mAbs (4, 5, 12–20). Third, polyclonal antibodies produced in their Fab’2 format from horses (21) or in their IgG format from humanized cows (22) or glyco-humanized pigs (23) have also proven efficacy to neutralize SARS-CoV-2. The safety and tolerability in humans of Fab’2 from horses and of humanized IgG polyclonal antibodies have been confirmed recently in different clinical trials (Lopardo et al., 2021 (21), NCT04453384, NCT04469179, Gaborit et al., 2021 (24)), contrasting with unmodified polyclonal antibodies containing wild-type IgG antibodies that induce serum sickness and allergic reactions (including fever and skin rashes) in 20% to 30% of the patients, excepting for those who concomitantly receive immunosuppression and high doses of steroids (25, 26). A partial efficacy of anti-SARS-CoV-2 Fab’2 from horses has been reported in those patients with negative baseline antibodies (NCT04494984). The efficacy of humanized or glyco-humanized IgG polyclonal antibodies in COVID-19 is still being investigated (NCT04453384, NCT04928430). Glyco-humanized swine antibodies are novel therapeutic modalities launched in 2019 after immunization of alpha 1,3-galactosyltransferase (GGTA1)/cytidine monophosphate-N-acetylneuraminic acid hydroxylase (CMAH) double KO pigs. Absence of the two corresponding alpha-Gal and Neu5GC xenoantigens has been shown to prevent the formation of immune complexes with human natural antibodies and is meant to avoid post-infusion serum sickness and allergies (23). Glyco-humanized swine antibodies against human T cells have already been used as an induction treatment in kidney transplant patients in the frame of a clinical phase 2 trial (ClinicalTrials.gov Identifier: NCT04431219).

The possible advantage of polyclonal antibodies over mAbs is their recognition of an array of epitopes on the target antigen, which should theoretically be not or less affected by antigen variations. Here, we investigated the extent to which XAV-19, a glyco-humanized swine polyclonal antibody previously shown to present neutralizing activity against SARS-CoV-2 Wuhan and D614G (B.1, PANGOLIN lineage) viruses (23), reduces viral load *in vivo* in human ACE-2-expressing mice and binds multiple target epitopes on SARS-CoV-2 Spike, and whether it maintains activity against the United Kingdom (Alpha/B.1.1.7), South African (Beta/B.1.351), Brazil (Gamma/P.1), and Indian (Delta/B.1.617.2) variants of concern.

## Methods

### Reagents

XAV-19 is a swine glyco-humanized polyclonal antibody against SARS-CoV-2 obtained by immunization of pigs with double knockout for alpha 1,3-galactosyltransferase (GGTA1) and cytidine monophosphate N-acetyl hydroxylase (CMAH) genes, as previously described (23). It corresponds to the whole IgG fraction, including all possible IgG subclasses, extracted from swine immune serum by capture and polishing chromatography. Intermediate R&D preparations of swine glyco-humanized polyclonal antibody against SARS-CoV-2 had been generated, presenting variable anti-SARS-CoV-2 binding activities (23). XAV-19 batches used in this study were clinical batches BMG170-B02, B03, and B06, which showed comparability in release testing. Comparator bamlanivimab is from Lilly (Indianapolis, IN, USA). Recombinant Spike molecules of the Wuhan type (Sino Biological ref 40591-V08H), mutation-containing RBD (Y453F ref 40592-V08H80; N501Y, ref 40592-V08H82; N439K, ref 40592-V08H14; E484K, ref 40592-V08H84), Alpha (ref 40591-V08H12; containing mutations HV69-70 deletion, Y144 deletion, N501Y, A570D, D614G, P681H), and Beta (ref 40591-V08H10; containing mutations K417N, E484K, N501Y, D614G) forms and recombinant human Fc-tagged ACE-2 were purchased by Sino Biological Europe, Eschborn, Germany.

SARS-CoV-2 Wuhan (D614 and D614G B.1variant), Alpha, Beta, Gamma, and Delta strains were isolated from SARS-CoV-2-infected patients in the Pitié-Salpêtrière, Aix-Marseille, and Toulouse University hospitals (France). The BetaCoV/Hong Kong/VM20001061/2020 [HK1] Wuhan was isolated at The Chinese University of Hong Kong (China).

### Binding ELISA

The target antigen (SARS-CoV-2 Spike RBD-HIS protein, Sino Biological Europe) was immobilized on Maxisorp plates at 1 µg/ml in carbonate/bicarbonate buffer at 4°C overnight. After washing, saturation was performed with PBS-Tween-BSA for 2 h at room temperature. Samples were diluted into PBS-Tween and added into the plate in duplicate, incubated 2 h at RT, and washed three times. Bound pig IgGs were revealed with a secondary anti-pig-HRP-conjugated antibody (Bethyl Laboratories, USA) diluted in washing buffer, at 1:1,000, incubated 1 h at RT, and washed three times. TMB reagent was added in the plate, incubated up to 20 min in the dark, and the reaction was stopped with H_2_SO_4_. Reading was performed at 450 nm.

### Spike/ACE-2 Neutralization Assay

An ELISA assay was developed to assess the properties of anti-SARS-CoV-2 Spike antibodies to inhibit the binding of ACE-2 to immobilized Spike. SARS-CoV-2 Spike S1-HIS (Sino Biological Europe; either Wuhan, Alpha or Beta) was immobilized on Maxisorp plates at 1 µg/ml in carbonate/bicarbonate buffer pH 9.0 at 4°C overnight. The plates were washed in PBS-Tween-0.05% and saturated with PBS-Tween-0.05%-2% skimmed milk for 2 h at room temperature (RT). Anti-Spike RBD antibodies diluted in PBS-Tween-0.05%-1% skimmed milk were then added and incubated for 30 min. Then, ligand human ACE-2-mFc tag (Sino Biological; 125 ng/ml final concentration) was added in the same dilution buffer. After 1-h incubation at room temperature and three washes, the mouse Fc tag was revealed with a specific HRP-conjugated antimouse IgG secondary antibody (diluted in in PBS-Tween-0.05%-1% skimmed milk powder at 1:1,000, incubated 1 h at RT, and washed three times). TMB reagent was added into the plate, incubated 6 min in the dark, and the reaction was stopped with 50 µl, 1 M H_2_SO_4_. The plate was read at 450 nm.

### Determination of XAV-19 Target Epitopes

A peptide microarray analysis has been performed using the PepStar™ system (JPT Peptide Technologies, Berlin, Germany). A total of 53 purified synthetic 15-meric overlapping peptides derived from RBD (sequence from BDSOURCE accession number NC_045512.2), with an additional C-terminal glycine (added for technical reasons), were covalently immobilized on glass surface. Full-length human IgG, mouse IgG, and pre-immune pig IgG were co-immobilized on microarray slides as assay controls. The XAV-19 sample used in the analysis is the clinical drug substance batch BMG170-B06, hybridized at the dilution of 100 µg/ml (the same applied for control swine IgG) for 1 h at 30°C on microarray slides. After sample incubation, secondary fluorescently labeled mouse anti-pig-IgG antibody diluted 1:5,000 was added in the corresponding wells and left to react for 1 h. Finally, a tertiary fluorescently labeled antimouse-IgG antibody at 1 μg/ml was incubated for 1 h to detect bound anti-pig-IgG secondary antibody. After washing and drying, the slides were scanned with a high-resolution laser scanner at 635 nm to obtain fluorescence intensity profiles. The slides were scanned with a receiver gain of 900 V, and images were quantified to yield a mean pixel value for each peptide.

The proteolytic epitope mapping comprised a proteolytic digestion step of the RBD protein, the isolation of resulting peptides by XAV-19 affinity chromatography, and the LC-MS/MS analysis of the eluted peptides. In short, the RBD protein was reduced, alkylated, and digested with an enzyme/protein ratio of 1:50 during 3 h at 37°C with the endopeptidases trypsin, chymotrypsin, or Arg-C. Digestion products were immunocaptured on Sepharose 4B on which XAV-19 IgG has been immobilized during 2 h at room temperature. Columns were then washed (ammonium bicarbonate 25 mM), and elution was performed with a Glycine/HCl 50 mM pH 2 buffer. Eluted fractions were then resolved by C18 inverted phase chromatography and tandem analyzed (MS/MS) to measure peptide masses. Data were compared with theoretical masses resulting from an *in silico* RBD digestion with the corresponding enzyme.

### Cytopathogenic Effect (CPE) Assay

Vero cells (CCL-81) and Vero E6 cells (CRL-1586) were obtained from the American Type Culture Collection and maintained at 37°C with 5% CO_2_ in Dulbecco’s Modified Eagle’s Medium (DMEM), supplemented with 5% heat-inactivated fetal bovine serum (FBS) and 1X penicillin-streptomycin solution (Thermo Fisher Scientific, USA). SARS-CoV-2 clinical isolates (D614G variant; GenBank accession number MW322968), Alpha (GenBank accession number MW633280), Beta (GenBank accession number MW580244), Gamma (Gene accession number pending), and Delta (Gene accession number pending) were isolated from SARS-CoV-2 RT-PCR confirmed patients by inoculating Vero cells with a sputum sample or nasopharyngeal swabs in the biosafety level-3 (BSL-3) facility of the Pitié-Salpêtrière University Hospital. Viral stocks were generated using one passage of isolates on Vero cells. Titration of viral stock was performed on Vero E6 by the limiting dilution assay allowing the calculation of tissue culture infective dose 50% (TCID50). The neutralizing activity of XAV-19 was assessed with a whole virus replication assay using the five SARS-CoV-2 isolates. XAV-19 was subjected to serial twofold dilution ranging from 50 to 0.05 µg/ml in fresh medium. About 50 µl of these dilutions was incubated with 50 µl of diluted virus (2 x 10^3^ TCID_50_/ml) per well in a 96-well plate at 37°C for 60 min in eight replicates. A hundred microliters of a Vero E6 cell suspension (3 x 10^5^ cells/ml) was then added to the mixture and incubated at 37°C under an atmosphere containing 5% CO_2_ until microscopy examination on day 4 to assess CPE. An infectivity score has been assigned on each well: 0, no cytopathic effect; 1, a fraction of cells was affected; and 2, 100% cells affected. The addition of the scores in the eight replicates was then transformed in the percentage of the maximal scoring (e.g., score of 16 = 100%). For viral load (VL) quantification, a similar experiment was conducted with a range of XAV-19 dilution ranging from 24 to 1 µg/ml in fresh medium. On day 4, RNA extraction of the eight pooled replicates of each XAV-19 dilution was performed with NucliSENS EasyMag (BioMerieux) according to the manufacturer’s protocol. The relative VLs were assessed from cycle threshold values for the ORF1ab gene obtained by the TaqPath™ COVID-19 RT-PCR (ThermoFisher, Waltham, USA) and by linear regression in log10 copies/ml with a standard curve realized from a SARS-CoV-2 positive nasopharyngeal sample quantified by Droplet-Digital PCR (Bio-Rad). IC50s were analyzed by nonlinear regression using a four-parameter dosage-response variable slope model with the GraphPad Prism 8.0.2 software (GraphPad Software, USA). To further analyze the neutralization potency and to confirm data with other independent laboratories, plaque reduction neutralization tests (PRNT)/CPE assays and viral load evaluation were carried out independently on Vero E6 cells at the BSL-3 facility of VibioSphen, University Paul Sabatier, Toulouse, France and of Aix-Marseille University, Marseille, France. SARS-CoV-2 Wuhan, Alpha, and Beta strains were isolated from patients with laboratory-confirmed COVID-19 from the corresponding university hospital. The viral isolates were amplified by one additional passage in Vero E6 cells to make working stocks of the virus. Vero E6 cells were cultured in Dulbecco’s modified Eagle’s medium (DMEM) supplemented with 10% v/v fetal bovine serum and 1% v/v penicillin-streptomycin supplemented with 1% v/v sodium pyruvate at 1 x 10^5^ cells per well in 12-well tissue culture plates. At 100% confluence (2 days post-seeding), the cells were washed twice with PBS, and six serial dilutions of the virus (1/10 each time) were added to the cells. Following infection with 0.3 ml per well of each dilution, plates were incubated at 37°C for 1 h, and the cells were washed with PBS before the addition of 2% w/v agar containing 1 μg/ml-5 tosyl phenylalanyl chloromethyl ketone-trypsin (Sigma-Aldrich) to the cell surface. Plates were left at room temperature for 20–30 min to allow for the overlay to set and were then incubated at 37°C for 72 h. Cells were fixed with 4% v/v paraformaldehyde before both fixative and agar were removed, and cells were stained with 0.1% w/v Crystal Violet (Fisher) in 20% v/v ethanol. Plaque titers were determined as plaque forming units per ml. CPE reduction assay was performed as follows: Vero E6 cells were seeded in 96-well clusters at a density of 5,000 cells/well 2 days before infection. Twofold serial dilutions, starting from 100 µg/ml of XAV-19, were mixed with an equal volume of a viral solution containing 300 pfu of SARS-CoV-2 (final volume 200 μl). The serum–virus mixture was incubated for 1 h at 37°C in a humidified atmosphere with 5% CO_2_. After incubation, 100 μl of each dilution was added in eight wells of a cell plate containing a semiconfluent Vero E6 cell monolayer. Control cells were infected with COVID-19 at MOI 0.01. Remdesivir (25 µM) was used as a positive control. After 3 days of incubation, the plates were inspected by an inverted optical microscope. Viable cells were quantified with CellTiter-Glo 2.0 luminescent cell viability assay.

### Antibody Escape Study

XAV-19 was subjected to serial twofold dilutions ranging from 50 to 0.2 µg/ml in fresh medium. Fifty microliters of XAV-19 dilutions was incubated with 50 µl of diluted virus (2 x 10^3^ TCID_50_/ml) per well in a 96-well plate at 37°C for 60 min in duplicates. A hundred microliters of a Vero E6 cell suspension (3 x 10^5^ cells/ml) was then added to the mixture and incubated at 37°C under an atmosphere containing 5% CO_2_. A no-antibody control was included to account for any cell culture adaptations of each SARS-CoV-2 variant. Virus replication was monitored on day 4 by screening for cytopathic effect. The supernatants were collected from wells with the highest antibody concentration displaying evident CPE. Fifty microliters of supernatants was used to infect new Vero E6 cells to which greater fresh XAV-19 concentrations were added. This procedure was repeated over five passages. RNA extraction was also performed on these supernatants with NucliSENS EasyMag (BioMerieux) according to the manufacturer’s protocol. Sanger sequencing was performed on the last passage (fifth) of each variant.

### Human ACE-2 Mouse Model

The protocol of the animal experiments was described in a previous study (27). Balb/c mice were first infected with 10^8^ TCID_50_ of the adenovirus carrying human ACE-2 protein intranasally. After 5 days post-infection, mice received intranasal administration of 10^5^ PFU of SARS-CoV-2 (BetaCoV/Hong Kong/VM20001061/2020 [HK1]). XAV-19 was administrated by intraperitoneal injection (I.P.) 24 h before or after the infection. Lungs were collected at day 3 post-infection, at a time where the viral load was maximal in this model (27), and the viral load was measured by tissue culture infectious dose (TCID_50_) using Vero E6 cells. The animal experiments were performed in the BSL3 facility of the University of Hong Kong. The study protocol was carried out in strict accordance with the recommendations and was approved by the Committee on the Use of Live Animals in Teaching and Research of the University of Hong Kong (CULATR 5499-20).

## Results

### XAV-19 Binding to SARS-CoV-2 Spike Correlates With Neutralizing Potency

**Figure 1:**
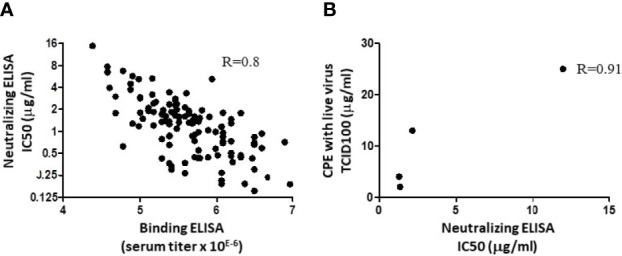
Correlation between binding, neutralizing ELISA, and CPE assay. **(A)** A series of 117 individual hyperimmune serum samples were assessed in parallel in an RBD binding ELISA and in a Spike/ACE-2 interaction ELISA. Linear correlation was observed with R= 0.8. Dots represent data from duplicate measurements in a single experiment. **(B)** Four R&D batches of swine anti-RBD polyclonal IgG were produced, with different binding activities against SARS-CoV-2 Spike (23). These samples were evaluated in parallel in the Spike/ACE-2 neutralization assay, as in A, and by CPE. IC50 in ELISA and CPE, expressed in tissue culture infectious dose (TCID_100_), are from single experiments and have been plotted to evaluate the correlation. The R value after linear extrapolation was 0.91.

Antibodies elicited after immunization with SARS-CoV-2 Spike can bind to their target and optionally present neutralizing activity. To assess whether binding intensity correlates with the neutralization potency, a series (n=117) of individual R&D serum samples drawn from immunized animals at different timepoints were evaluated in parallel in a binding ELISA and in a Spike/ACE-2 neutralization ELISA. The data (**Figure 1A**) indicated that the binding assay (expressed in serum titer) correlated with the neutralization assay (IC50, inhibitory concentration to inhibit 50% of the signal; R=0.8). The next question was whether analyzing neutralization with a Spike/ACE-2 neutralization ELISA predicts the neutralization of cell infection using live viruses. To answer the question, four R&D anti-SARS-CoV-2 IgG batches made from pooled serum samples (different from those presented in **Figure 1A**) presenting variable anti-RBD binding activities were assessed in parallel in neutralizing ELISA and in CPE assays. The batches presented IC50 values by ELISA of 1.3, 1.34, 2.2, and 12 µg/ml, and the corresponding values in CPE assays were of 3, 2, 12.5, and 25 µg/ml, thus also showing a correlation (R=0.91; **Figure 1B**). For further analyses and clinical use, GMP batches are similar to the R&D batches presenting the highest activity (lower left dots in **Figure 1B**).

### XAV-19 Targets Multiple Epitopes on RBD

**Figure 2:**
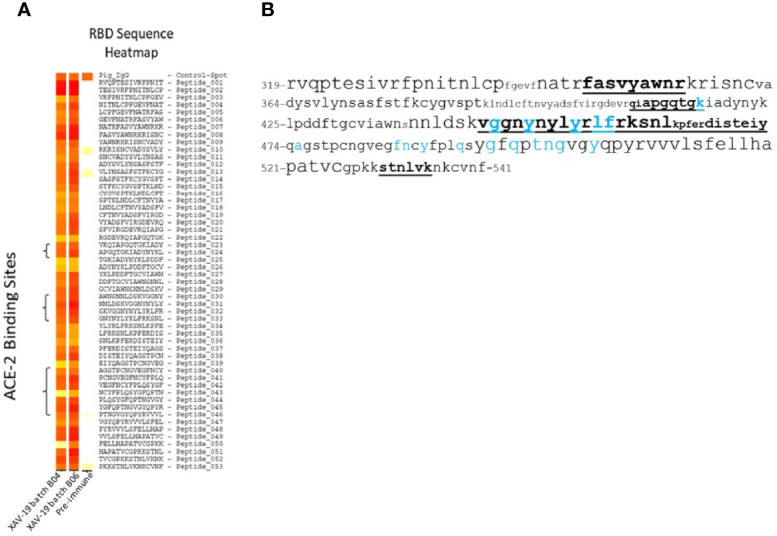
XAV-19 target epitopes. **(A)** Heatmap from a peptide array experiment showing the binding intensity of two XAV-19 batches and pre-immune IgG to 15-meric peptides of the RBD sequence, overlapping by 10 amino acids. White, no binding; yellow, background binding; light red, medium binding; dark red, strong binding. Positive control: pig IgG spotted on the slides (Control-Spot). Negative controls, human and mouse IgG spotted on the slides, were negative (not shown). **(B)** XAV-19 target epitopes on the RBD domain (amino acid sequence numbered according to DBSOURCE sequence reference NC_045512.2) as recorded in LC-MS/MS peptide mapping. Font size refers to binding intensity in the peptide array shown in A: font 10, weak recognition; font 14, medium recognition; font 18, strong recognition. Underlined bold: XAV-19 target peptides as defined in LC-MS/MS. Blue characters: amino acids in contact with ACE-2, according to Jafary et al. (28).

Two orthogonal methods have been used to identify the epitopes recognized by XAV-19 on the RBD protein. First, all 15-meric peptides overlapping by 10 amino acids (thus a total of 53 peptides) were spotted on glass slides and hybridized with two GMP batches of XAV-19 or pre-immune swine GH-pAb control antibodies. The resulting heatmap plot revealed that all peptides, though to varying extents, could be specifically recognized by antibodies contained in XAV-19 (**Figure 2A**). Since XAV-19 antibodies cannot bind all peptides simultaneously when contained in the Spike protein, for steric hindrance, a second investigation was undertaken to identify which peptides in the RBD domain in a more native configuration are recognized by XAV-19 antibodies. The assay was based on the recognition by XAV-19 antibodies of RBD-derived peptides obtained by proteolytic digestion (three enzymes tested: trypsin, chymotrypsin, and Arginase-C) and an isolating step of the resulting peptides by affinity chromatography (XAV-19 being immobilized on Sepharose) followed by an LC-MS/MS analysis of the eluted peptides. This proteolytic epitope mapping analysis revealed several recognition areas on the RBD protein (**Supplementary Table 1**). The peptides where the two methods gave the strongest overlapping hits, thus most probably representing dominant target epitopes, were amino acids 347–355 and 445–461. Amino acids 409–417, 462–473, and 530–535 were also found to be protected in the LC-MS/MS analysis, although less recognized in the peptide array (**Figure 2B**). Interestingly, six amino acids described to directly interact with human ACE-2 (28) are located in these regions.

### Neutralization of Wuhan, Alpha, Beta, Gamma, and Delta SARS-CoV-2 Variants

**Figure 3:**
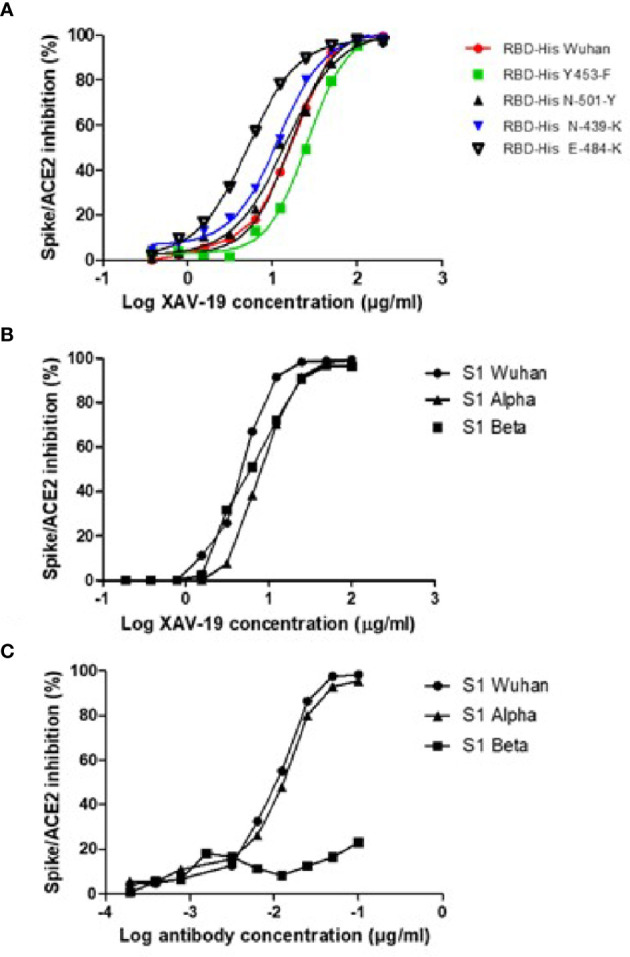
Neutralization assay in the ELISA format: assessment of SARS-CoV-2 Spike/ACE-2 interaction and its anti-RBD antibody-mediated inhibition. Spike-HIS containing the indicated mutations **(A)** or grouped mutations corresponding to the Alpha and Beta variants **(B, C)** was immobilized on plastic, and binding of recombinant human ACE2-Fc was revealed with a secondary antibody against Fc. 100% inhibition represents absence of Spike/ACE-2 interaction. **(A, B)** Means of triplicate measurements run in a single experiment assessing XAV-19 at the indicated concentration. **(C)** Means of triplicate measurements run in a single experiment assessing bamlanivimab at the indicated concentration.

XAV-19 was tested in a Spike/ACE-2 binding competition assay, where the Spike protein was of the original Wuhan type or contained the RBD mutations N501Y, N439K, and Y453F described in the Alpha and Beta variants, or the mutation E484K to induce resistance to mAbs (29). Variants expressing a combination of mutations present in the Spike Alpha (HV69-70 deletion, Y144 deletion, N501Y, A570D, D614G, P681H) or Beta (K417N, E484K, N501Y, D614G) were also tested. All single mutation forms of the Spike could be fully neutralized at concentrations not significantly different (slightly lower for the E484K mutation) from the Wuhan type (**Figure 3A**). XAV-19 also demonstrated a 100% inhibitory activity on the two Spike proteins fully representative of the Alpha and Beta variants, similar to the Wuhan Spike, with IC50 values of 6.4, 4.0, and 4.5 µg/ml, respectively (**Figure 3B**). Bamlanivimab, tested in parallel, demonstrated a potent inhibitory capacity against the Wuhan and Alpha variants, with an IC50 value of 0.01 µg/ml but, as described (30), failed to inhibit the binding of Beta Spike to ACE-2, even at a high concentration (**Figure 3C**).

**Figure 4:**
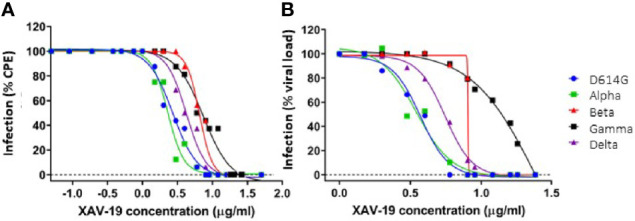
*In vitro* neutralization of SARS-CoV-2 variants by XAV-19. XAV-19 neutralizing potency was evaluated in an *in vitro* assay using whole replicating viruses. Percentage of infection was evaluated as described in *Methods*, based on cytopathogenic effect (CPE) **(A)** and virus RNA load **(B)** after infection with SARS-CoV-2 viruses of the indicated variants. CPE percentage was assessed by microscopy examination and calculated on eight replicates for each XAV-19 concentration. 100% represents the absence of CPE inhibition at the studied concentration, as found in control (no inhibitor) condition. Viral load percentage was calculated as the ratio of the viral load in each XAV-19 concentration to the viral load in controls (no inhibitor). XAV-19 concentrations are expressed on a 10 logarithmic scale. Blue dot: D614G/B.1 variant; green square: Alpha/UK/B.1.1.7 variant; red triangle: Beta/SA/B.1.351 variant; black square: Gamma/BR/P.1 variant; purple triangle: Delta/B.1.617.2 variant.

To further determine the neutralizing effect of XAV-19 using live viruses, CPE assays were performed in three different platforms (Paris Sorbonne University, Aix-Marseille University, and Paul Sabatier Toulouse University) using Wuhan (D614), B.1 (D614G PANGOLIN lineage) (D614G), Alpha, Beta, Gamma, and Delta SARS-CoV-2 clinical isolates, as previously described (31). The test assessed the inhibition of live viruses with sensitive Vero E6 cells and recorded infection after 4 days by assessing CPE and viral load by RT-qPCR. Data showed similar neutralizing potency for the Wuhan B.1 (D614G), Alpha, and Beta strains in a first set of experiments (**Supplementary Figure 1**) and showed global similar potency of XAV-19 on the Wuhan D614G, Alpha, Beta, Gamma, and Delta strains in a second set of experiments (**Figure 4**), with the absence of neutralizing activity below 1.5 µg/ml and 100% neutralizing activity above 5 to 10 µg/ml. **Table 1** shows recorded IC50 values assessed in one to three independent experiments in different laboratories for up to three GMP batches of XAV-19 and bamlanivimab assessed in parallel.

**Table 1:** Potency assessment of XAV-19 (GMP drug substance batch B03/04) by CPE and RT-PCR quantification of residual viral RNA after infection of Vero E6 cells with virus variants.

IC50 CPE (ng/ml)	Wuhan D614	Wuhan G614	Alpha	Beta	Gamma	Delta
XAV-19 batch B01	ND	6250	ND	ND	ND	ND
XAV-19 batch B02	6250	3130	ND	ND	ND	ND
XAV-19 batch B03/04	400; 3130; 3130	2722; 2208; 2720	100; 2208; 2292	6560; 3226	8196	4226
Bamlanivimab	ND	103; 84	108; 100	>50,000; >50,000	ND	ND
IC50 viral RNA inhibition (ng/ml)	Wuhan D614	Wuhan G614	Alpha	Beta	Gamma	Delta
XAV-19 batch B03/04	392	584; 3733; 3681	408; 1265; 3482; 3590	8057; 8933	13880	5557
XAV-19 batch B06	312	593	523; 532	1157	721	646
Bamlanivimab	ND	117	486	>50,000	ND	ND

### Absence of XAV-19 Induced SARS-CoV-2 Mutation *In Vitro*

Antibodies, by applying a selective selection pressure, can favor the outgrowth of resistant novel variants with unknown properties, especially in conditions where their neutralizing potency is suboptimal. To investigate whether XAV-19 is susceptible to generate such variants, Vero E6 cells were infected with 100 TCID50/50 µl of either B.1D614G, Alpha, Beta, Gamma, or Delta strains and maintained over five passages (20 days) with culture medium or culture medium containing increasing concentrations of XAV-19. After passage 5, the emergence of antibody escape mutants was evaluated by Sanger sequencing. The data from the sequencing are summarized in **Table 2**. They show absence of mutations in the RBD domain (amino acids 331 to 524) under any condition. Variations were found in the Spike outside the RBD domain, not associated with the addition of XAV-19. Overall, 11 mutations were found without antibody addition (culture medium only), whereas 5 mutations were found with XAV-19. These mutations probably represent adaptations to the culture conditions. None of these mutations has been described as resistant to antibodies.

**Table 2:** Amino acid substitutions in the SARS-CoV-2 Spike detected after five passages in Vero E6 cells with or without addition of XAV-19.

	Bav Pat D614G	Alpha	Beta	Gamma	Delta
No XAV-19 control	G72R/G, D215N/DS1249F/S	S151R	H66R	Ø	M153R/M S686R
XAV-19 increasing concentration	S1170F	S151R	H66H/R	S813I/S	Ø

### XAV-19 Reduces Lung Viral Load in Human ACE-2 Expressing Mice

**Figure 5:**
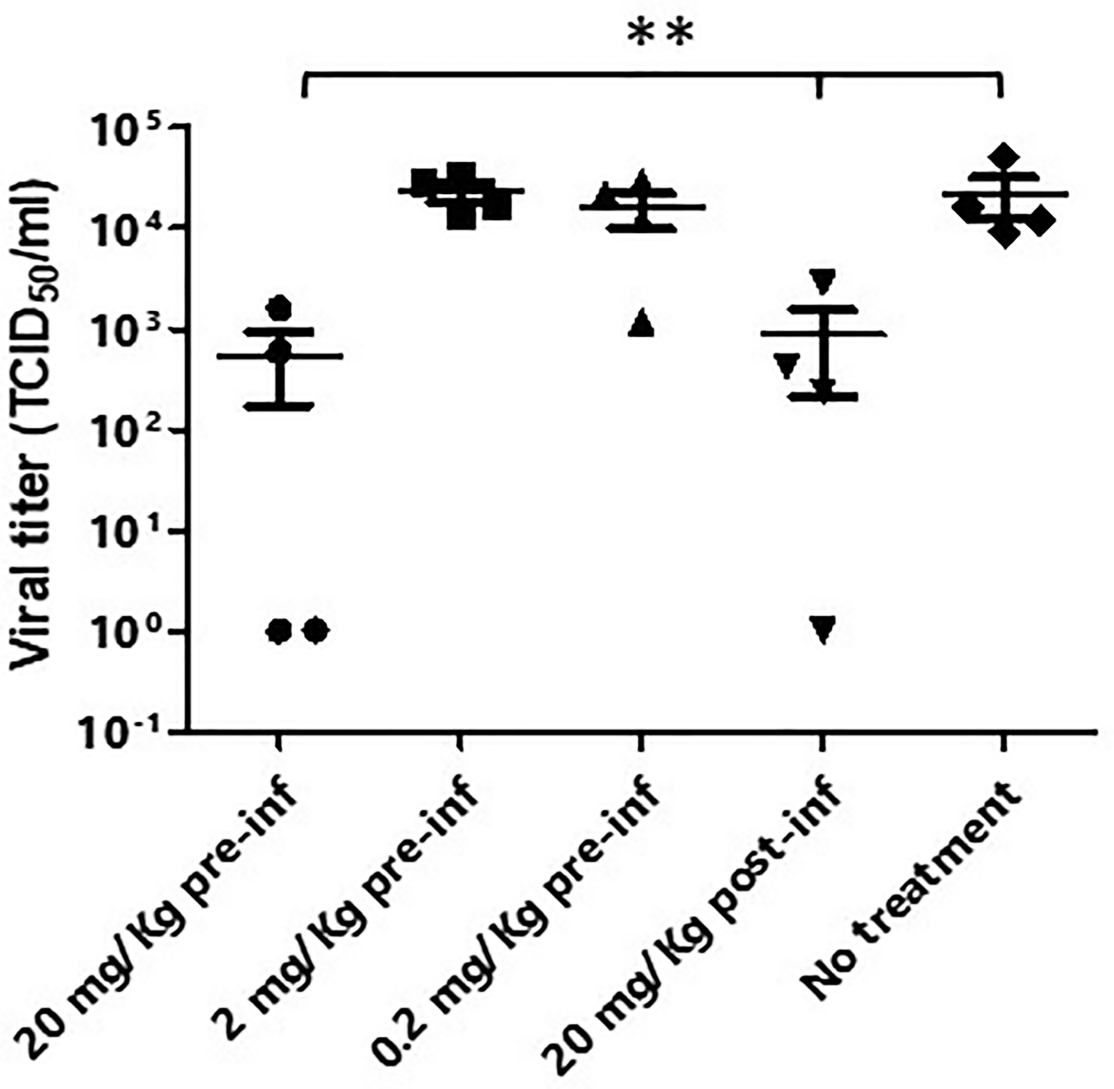
Murine model of SARS-CoV-2 infection. Balb/c mice (n=4/group) were first transduced intranasally with adenovirus coding for human ACE-2. After 5 days, mice were infected intranasally with SARS-CoV-2 (Wuhan strain) and received the indicated dose of XAV-19, intraperitoneally, 24 h before (pre-inf) or after (post-inf) infection. Three days later, lungs were necropsied and homogenized and the viral load was evaluated by PRNT. Each dot represents a single mouse and is the mean of eight replicates assessed in a single experiment. Nonparametric statistics were used for pairwise comparisons using Kruskal–Wallis tests. **p < 0.01.

Balb/c mice with human ACE-2 expression in the lung were infected with 10^5^ PFU SARS-CoV-2 intranasally. XAV-19 was administrated through intraperitoneal injection under different experimental conditions: 1) 20 mg/kg, 24 h before viral infection; 2) 2 mg/kg, 24 h before viral infection; 3) 0.2 mg/kg, 24 h before viral infection; 4) 20 mg/kg, 24 h after viral infection; and 5) untreated. The results showed a 98% reduction of the viral load in the lung at day 3 if 20 mg/kg of XAV-19 was given to the mice 24 h before the infection and a 94% reduction if 20 mg/kg of XAV-19 was given 24 h after the infection. No significant reduction of the viral load was observed from the group of administrating either 2 or 0.2 mg/kg 24 h before infection (**Figure 5**). Whatever the treatment group, no virus was found anymore on day 5 post-infection (data not shown).

## Discussion

XAV-19 is a swine glyco-humanized neutralizing polyclonal antibody raised against the RBD protein of the SARS-CoV-2 original Wuhan strain (17). In this paper, we report that XAV-19 also fully neutralizes Alpha (United Kingdom/B.1.1.7), Beta (South African/B.1.351), Gamma (Brazil/P.1), and Delta (Indian/B.1.617.2) variants *in vitro*. Because XAV-19 is manufactured after pooling many individual serum samples drawn on multiple donor animals, it presumably contains a diversity of IgG molecules presenting an array of avidities and seeing several epitopes on the target protein. When analyzing individual sera (from individual donor animals) composing XAV-19, a correlation was observed between binding and neutralizing activities. This indicated that globally, most antibodies in XAV-19 bind to RBD in a way that prevents SARS-CoV-2 Spike interaction with human ACE-2. Because the RBD immunization antigen and the Spike-HIS protein used in neutralizing assays are recombinant molecules, it was important to control that the corresponding RBD domain in live viruses could also be similarly targeted and neutralized. Parallel testing of four XAV-19 R&D batches presenting with varying neutralization potencies confirmed that a high neutralization potential assessed *in vitro* with a recombinant Spike-Fc molecule corresponded to a high neutralization potential in cytopathic assays using live viruses. This observation confirmed the data showing that a neutralizing ELISA is predictive of SARS-Cov-2 neutralization assessed in a lentivirus-pseudotyped SARS-CoV-2 neutralization assay (4).

Target epitopes of XAV-19 on RBD differed whether assessed by peptide microarray or by proteolytic peptide mapping. Peptide microarrays indicated that all possible 15-meric peptides contained in the RBD protein have been immunogenic in animals and generated specific antibodies. In a real situation, however, not all antibodies can be engaged together, and some of them might fail to get access to the corresponding protein domain, for steric hindrance. The proteolytic peptide mapping assay allows the presentation of large peptides issued from RBD proteolysis and therefore is believed to be more relevant. The proteolytic peptide mapping partially confirmed data from the peptide microarrays and identified several target epitopes containing amino acids important for the interaction between Spike and human ACE-2, explaining the neutralizing activity of XAV-19. The Alpha, Beta, Gamma, and Delta variants contain mutations in the RBD domain, which might impact recognition by anti-RBD antibodies. K417N/T and E484K are present in Beta and Gamma variants, N501Y is present in the Alpha, Beta, and Gamma variants, and T478K appears in the Delta variant. These mutations are located in peptides scored as “medium” in the peptide microarray and not revealed by proteolytic peptide mapping. These mutations might therefore have no or a limited impact on XAV-19 binding. In contrast, L452R is present in the Delta variant and is located in a region seen as a major target epitope by both techniques. This predicts that antibodies binding to the corresponding peptide would be affected. However, XAV-19 behaves similarly toward the Delta variant, with even a stronger neutralization of the viral load after infection of Vero E6 cells (**Figure 4B**). It is possible that in a polyclonal antibody, although a specific antibody is prevented from recognizing its target (because of a change in the epitope), other antibodies remain able to bind a closely related epitope. This is what is suggested by the peptide array data showing many binding possibilities.

Independent cytopathic assays with live viruses were run in parallel in different locations by different teams. They show similar findings, i.e., that a concentration of XAV-19 up to 10 µg/ml is required to fully neutralize all the variants. There was a clear tendency of higher concentration required to exhibit similar neutralizing capacity against the Beta and Gamma variants, as compared with Wuhan, Alpha, and Delta forms. This difference was observed in different assays, whether measuring target cell viability, cytopathogenic effect, or viral RNA load, and confirmed in different experiments, thus reflecting actual differences rather than experimental variability. However, important differences in IC50 values were observed for neutralization of Beta, Gamma, and Delta variants using two different XAV-19 batches (**Table 1**). Here, we cannot exclude experimental interplatform variability since the two different XAV-19 batches used presented potencies considered as comparable using a good laboratory practice-validated ELISA method in release testing. Thus, our results demonstrate that XAV-19 can fully neutralize all SARS-CoV-2 variants, and a clear difference was evident when compared to bamlanivimab, which has no neutralizing effect on the Beta variant.

When administered intraperitoneally to human-ACE-2 mice challenged intranasally with SARS-CoV-2 viruses, XAV-19 induced a dose-dependent reduction of the viral load in the lung, demonstrating the potential to localize to infected tissues. The 94% to 98% reduction in the viral load noticed here is in agreement with the data obtained with different neutralizing antibodies in ferrets, hamster, or mouse models (32–40) or obtained with the REGN-CoV2 antibody cocktail in a comparable animal model (41). In a few studies, however, the viral reduction factor reached 3 or 4 log (33, 42–45). Interestingly, Gilliland et al. (22) reported the absence of viral reduction in the lung of SARS-CoV-2 challenged mice treated with a humanized cow polyclonal antibody, whereas a clear clinical impact was demonstrated. One limitation of our model in which mice airways are transduced with an adenovirus expressing human ACE-2 is the absence of clinical symptoms, as the mice eliminate the virus by themselves within a few days. The evaluation we made was restricted to the assessment of the viral load in the lung. Therefore, owing to the lack of correlation between the lung viral load and the clinical status, our data cannot inform whether XAV-19 can bring a clinical benefit or whether the 20 mg/kg dose found to be active in mice can be extrapolated for human use.

Early after the COVID-19 outbreak onset, many labs have been able to rapidly develop neutralizing antibodies. One year later, as of mid-2021, more than 93 clinical trials assessing the safety and benefit of mAbs and 8 of polyclonal antibodies are listed in the Clinicaltrials.gov repository (NCT04610502, NCT04838821, NCT04514302, NCT04834908, NCT04834089, NCT04518410, NCT04453384, and NCT04453384). In late 2020, variants of concern started to spread in the population and now cause the majority of infections. However, antibodies that are now assessed clinically have been mostly raised against the initial, Wuhan strain. It has therefore become essential to revisit their potential to also neutralize variants. XAV-19 is currently being tested in phase 2 and 3 studies (NCT04453384; Eudract Number: 2020-005979-12). Phase 2a demonstrated that a single intravenous perfusion of XAV-19 at 2 mg/kg was safe, achieving a median serum C_max_ of 50.4 µg/ml and day 8 concentration of 20.3 µg/ml with elimination half-life (T1/2) estimated at 11.4 days (24, 46). The data presented here, together with these pharmacokinetic data, indicate that XAV-19 can provide high and sustained therapeutic activity *in vivo*. These data warrant continuation of clinical studies with XAV-19, especially in a context where the Delta variant becomes dominant and other variants of concern emerge.

## Data Availability Statement

The raw data supporting the conclusions of this article will be made available by the authors, without undue reservation.

## Ethics Statement

The animal study was reviewed and approved by the Committee on the Use of Live Animals in Teaching and Research of the University of Hong Kong (CULATR 5499-20).

## Author Contributions

Conceived the study: OD and BV. Designed and supervised some experiments: OD, BV, RB, FR, SM, A-GM, and VC. Performed the experiments: GE, RS, A-GM, CC, P-JR, SM, IM, and CM. Analyzed the data: BV, OD, A-GM, GL, EL, EM, BG, and FR. All authors contributed to the article and approved the submitted version.

## Funding

This work was supported by Xenothera, the European Union’s Horizon 2020 research and innovation programme (grant agreement No 962036), the Agence Nationale de la Recherche sur le SIDA et les Maladies Infectieuses Emergentes (ANRS MIE), AC43 Medical Virology and Emergen Program, the SARS-CoV-2 Program of the Faculty of Medicine of Sorbonne Université and by Bpifrance, grant «Projet de Recherche et Développement Structurant Pour la Compétitivité spécifique à la crise sanitaire COVID-19—POLYCOR» and the National Research Foundation of Korea (NRF) grant funded through the Korea government (NRF-2018M3A9H4055203).

## Conflict of Interest

OD, P-JR, CC, GE, EL, and BV are employees of Xenothera, a company developing glycol-humanized polyclonal antibodies as those described in this manuscript.

The remaining authors declare that the research was conducted in the absence of any commercial or financial relationships that could be construed as a potential conflict of interest.

## Publisher’s Note

All claims expressed in this article are solely those of the authors and do not necessarily represent those of their affiliated organizations, or those of the publisher, the editors and the reviewers. Any product that may be evaluated in this article, or claim that may be made by its manufacturer, is not guaranteed or endorsed by the publisher.

## Supplementary Material

Supplementary Table 1 Epitope mapping by LC-MS/MS analysis. Peptides identified after digestion of the SARS-CoV-2 Spike target with either trypsin, chemotrypsin and Arg-C and then eluted from a XAV-19 immunoaffinity chromatography.
