# Corrigendum: XAV-19, a swine glyco-humanized polyclonal antibody against SARS-CoV-2 spike receptor-binding domain, targets multiple epitopes and broadly neutralizes variants

**DOI:** 10.3389/fimmu.2023.1208705

**Published:** 2023-04-28

**Authors:** Bernard Vanhove, Stéphane Marot, Ray T. So, Benjamin Gaborit, Gwénaëlle Evanno, Isabelle Malet, Guillaume Lafrogne, Edwige Mevel, Carine Ciron, Pierre-Joseph Royer, Elsa Lheriteau, François Raffi, Roberto Bruzzone, Chris Ka Pun Mok, Odile Duvaux, Anne-Geneviève Marcelin, Vincent Calvez

**Affiliations:** ^1^ Xenothera, Nantes, France; ^2^ Sorbonne Université, Institut National de la Santé et de la Recherche Médicale (INSERM) 1136, Institut Pierre Louis d’Epidémiologie et de Santé Publique (iPLESP), Assistance Publique-Hôpitaux de Paris (AP-HP), Pitié Salpêtrière Hospital, Department of Virology, Paris, France; ^3^ Hong Kong University (HKU)-Pasteur Research Pole, School of Public Health, Li Ka Shing (LKS) Faculty of Medicine, The University of Hong Kong, Hong Kong, Hong Kong SAR, China; ^4^ Department of Infectious Disease, Nantes University Hospital, Nantes, France; ^5^ Institut National de la Santé et de la Recherche Médicale (INSERM) CIC1413, Nantes University Hospital, Nantes, France; ^6^ Department of Cell Biology and Infection, Institut Pasteur, Paris, France; ^7^ Li Ka Shing Institute of Health Sciences, Faculty of Medicine, The Chinese University of Hong Kong, Hong Kong, Hong Kong SAR, China; ^8^ The Jockey Club School of Public Health and Primary Care, The Chinese University of Hong Kong, Hong Kong, Hong Kong SAR, China

**Keywords:** COVID-19, polyclonal antibody (PAb), SARS-CoV-2, variants, neutralization

In the published article, there was an error in the Funding statement within the Acknowledgements section (funding from the European Union was omitted). The Acknowledgments and Funding sections have now been split as well. The correct Acknowledgments and Funding statements appear below. 

