# Supplementary material for: XAV-19, a Swine Glyco-Humanized Polyclonal Antibody Against SARS-CoV-2 Spike Receptor-Binding Domain, Targets Multiple Epitopes and Broadly Neutralizes Variants
Source: Front Immunol. 2021 Nov 15;12:761250. doi: 10.3389/fimmu.2021.761250 (PMC8634597; doi:10.3389/fimmu.2021.761250)
Supplement: Supplementary Table 1 — Epitope mapping by LC-MS/MS analysis. Peptides identified after digestion of the SARS-CoV-2 Spike target with either trypsin, chemotrypsin and Arg-C and then eluted from a XAV-19 immunoaffinity chromatography. [file Table_1.docx]

**Supplementary Table 1: Epitope mapping by LC-MS/MS analysis. Peptides identified after digestion of the SARS-CoV-2 Spike target with either trypsin, chemotrypsin and Arg-C and then eluted from a XAV-19 immunoaffinity chromatography.**

| Peptides recognized by XAV-19 after tryspin digestion of RBD | Peptides recognized by XAV-19 after chemotryspin digestion of RBD | Peptides recognized by XAV-19 after Arg-C digestion of RBD |
| --- | --- | --- |
| \| fasvyawnr \| \| --- \| \| fasvyawnrk \| \| qiapgqtgk \| \| vggnynylyr \| \| ksnlkpfer \| \| snlkpfer \| \| stnlvk \| | \| rksnlkpf \| \| --- \| \| erdisteiy \| \| rvvvlsfellhapatvcgpkkstnl \| \| sfellhapatvcgpkkstnl \| \| hapatvcgpkkstnl \| | fasvyawnr |
