# Supplementary figures and images for: XAV-19, a Swine Glyco-Humanized Polyclonal Antibody Against SARS-CoV-2 Spike Receptor-Binding Domain, Targets Multiple Epitopes and Broadly Neutralizes Variants
Source: Front Immunol. 2021 Nov 15;12:761250. doi: 10.3389/fimmu.2021.761250 (PMC8634597; doi:10.3389/fimmu.2021.761250)

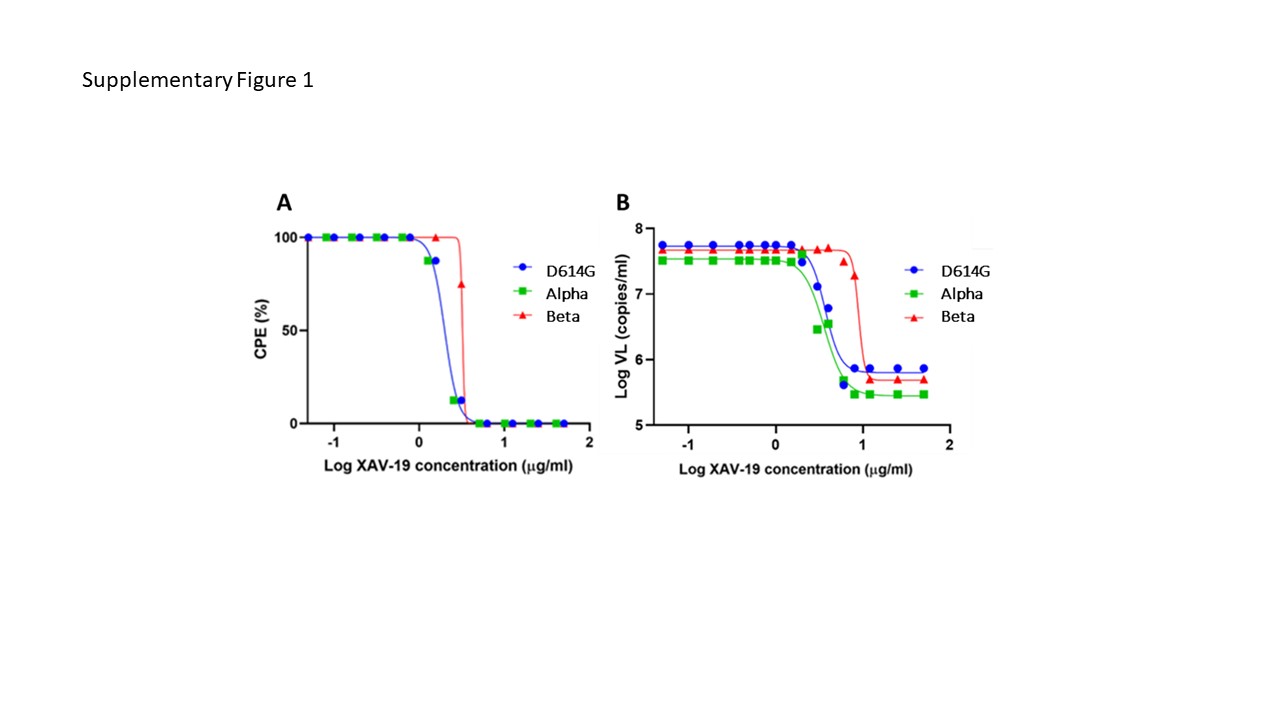

Supplement: Supplementary file 2 [file Image_1.jpeg]
